# Therapy Dogs at the Bedside: A Scoping Review of Pet Therapy in Adult Intensive Care Units

**DOI:** 10.7759/cureus.97092

**Published:** 2025-11-17

**Authors:** Joseph L Kim

**Affiliations:** 1 Emergency Medicine, University of Wisconsin School of Medicine and Public Health, Madison, USA

**Keywords:** animal assisted intervention, animal assisted therapy, icu, intensive care unit, patient experience, pet assisted intervention, pet therapy, therapy dogs

## Abstract

Adult intensive care unit (ICU) patients experience significant anxiety, distress, and isolation. Pet therapy or animal-assisted interventions, often delivered by certified therapy dogs, are used in hospitals to improve comfort and engagement, but ICU-specific evidence remains limited.

This scoping review maps the recent evidence on pet therapy in adult ICUs, with the aim of describing how it is used, what benefits are reported, what risks and barriers exist, and where the literature remains thin. Peer-reviewed publications from 2015 to 2025 were found through major biomedical databases (including PubMed, Cumulative Index to Nursing and Allied Health Literature (CINAHL), Embase, and Scopus) that reported live animal interactions in adult ICUs, along with any relevant reviews and implementation reports. The studies were independently screened according to the inclusion and exclusion criteria, and a total of 15 studies met eligibility criteria. Across the studies, patients showed immediate reductions in self-reported anxiety after visits, families often reported parallel relief, and satisfaction with the experience was high among patients, families, and staff. Pain scores improved modestly in some cohorts, while heart rate, blood pressure, and respiratory rate were generally unchanged, suggesting psychological benefit without physiologic destabilization. Pet therapy programs were feasible in the ICU when supported by clear policies and coordination, although common barriers included infection prevention concerns, scheduling and staffing needs, space constraints, and the limited availability of controlled data. The evidence was insufficient to determine effects on delirium, sedative exposure, length of stay, or mortality. Overall, pet therapy in adult ICUs appears feasible, well-accepted, and safe under structured protocols, with consistent short-term relief of anxiety and perceived distress and potential support for engagement in rehabilitation.

Future work should include multicenter controlled studies, formal safety surveillance, standardized outcome sets that include delirium and longer-term psychological recovery, cost and implementation analyses, and evaluation of robotic or virtual animal options when live visits are not possible.

## Introduction and background

Patients in adult intensive care units (ICUs) often endure significant physical and psychological stress. ICU survivors commonly describe ICU stays as traumatic, marked with confusion, anxiety, sleeplessness, pain, and loneliness [1]. Traditional management of these symptoms with sedatives and analgesics can cause harm. For example, excessive sedation is linked to delirium and worse outcomes [1]. These issues have prompted growing interest in non-pharmacological interventions to humanize the ICU environment and improve patient well-being [1]. Pet therapy, also known as animal-assisted intervention (AAI) or animal-assisted therapy (AAT), is one such approach. It involves guided interactions between patients and trained animals (usually but not always therapy dogs) for therapeutic benefit.

In general hospital and rehabilitation settings, animal-assisted therapy has shown promise in reducing anxiety and depression, easing pain, and improving mood and quality of life [1,2]. Therapy animals provide comfort and companionship that can alleviate emotional suffering and have been shown to help alleviate physical and emotional suffering patients may have [3]. For the benefits it brings, these observations are aligned with the broader push to humanize the ICU, treating the patient’s mind as well as body. However, because of how novel pet therapy is in an ICU setting, there are concerns such as infection control, animal behavior, allergies, and logistical barriers.

This scoping review synthesizes recent peer-reviewed literature (2018-2025) to provide a comprehensive review of pet therapy in adult ICUs, examining its applications, documented benefits, challenges, and the current state of evidence. Focus on physiological and emotional outcomes, physiological and clinical outcomes, and the feasibility, safety, and acceptability of pet therapy will be presented as the core structure of this scoping review. Exploration and discussion of the emerging evidence, psychological benefits, physical and rehabilitation outcomes, perspectives of the patient, family, and staff, and finally, the current utilization and program models will further serve as the framework, allowing the additional evidence to be presented clearly and progressively. Despite all the emerging research and findings, more robust studies are needed to establish the efficacy and safety of ICU pet therapy. In this context, pet therapy has emerged as a promising non-pharmacologic intervention, and in the interim, careful implementation of animal-assisted intervention that follow infection control guidelines and patient screening can offer a compassionate, holistic complement to ICU care.

## Review

Methodology

Review Question

The objective of this scoping review is to examine how pet therapy has been implemented and evaluated within adult ICUs, with particular attention to its effects on patient well-being and the overall ICU environment. Specifically, this review focuses on the following key areas: 1) the psychological and emotional outcomes associated with pet therapy among adult ICU patients, 2) the physiological and clinical effects observed in this population, and 3) the feasibility, safety, and acceptability of integrating pet therapy into adult ICU practice.

Study Design

The Joanna Briggs Institute (JBI) Scoping Review methodology was used to conduct this scoping review [4]. The review article was reported using the checklist from the Preferred Reporting Items for Systematic Reviews and Meta-Analyses extension for Scoping Reviews (PRISMA-ScR) [5].

Eligibility Criteria

For this scoping review, the JBI outline for the population-concept-context framework was used as a guideline. The articles included were published in peer-reviewed journals between 2015 and 2025. Eligible studies and reports examined adult ICU settings in which a live animal (or animal surrogate) was brought in for patient therapy or visitation. All study designs were considered, given the early evidence base, including randomized controlled trials, quasi-experimental studies, pre-post studies, observational cohorts, case reports, program evaluations, and qualitative reports. Inclusion required reporting outcomes, feasibility, or stakeholder perspectives related to pet therapy in an adult ICU, or in mixed acute-care settings with a substantial ICU component. Studies conducted solely in pediatric ICUs and studies limited to non-ICU hospital units were excluded unless they provided data clearly transferable to ICU contexts. Broader literature from rehabilitation or long-term care was used only for background when particularly influential. As this is a scoping review, a formal quality appraisal was not undertaken, and instead, the evidence is summarized narratively.

Information Sources

The databases used for this scoping review included PubMed, Cumulative Index to Nursing and Allied Health Literature (CINAHL), Embase, and Scopus.

Search Strategy

Examples of keywords included for the search strategy were "pet therapy," "animal-assisted intervention," "animal-assisted therapy," "therapy dog," "intensive care unit," "ICU," "adult ICU," and "humanization of care". Synonyms were also used. The search strategy is presented in Table 1.

**Table 1:** Search strategy

Database	Search Strategy
PubMed	("animal-assisted therapy"[MeSH] OR "animal assisted therapy"[tiab] OR "animal-assisted intervention*"[tiab] OR "pet therap*"[tiab] OR "therapy dog*"[tiab]) AND ( "Intensive Care Units"[MeSH] OR ICU[tiab] OR "intensive care"[tiab] OR "critical care"[tiab] ) AND ( "Adult"[MeSH] OR adult*[tiab] )
Embase	'animal assisted therapy'/exp OR 'animal assisted intervention':ab,ti OR 'pet therap*':ab,ti OR 'therapy dog*':ab,ti AND ('intensive care unit'/exp OR ICU:ab,ti OR 'intensive care':ab,ti OR 'critical care':ab,ti) AND ('adult'/exp OR adult*:ab,ti)
CINAHL	(MH "Animal Assisted Therapy+") OR TI ("animal-assisted" OR "pet therap*" OR "therapy dog*") OR AB ("animal-assisted" OR "pet therap*" OR "therapy dog*") AND (MH "Intensive Care Units+" OR TI (ICU OR "intensive care" OR "critical care") OR AB (ICU OR "intensive care" OR "critical care")) AND (MH "Adults+" OR TI adult* OR AB adult*)
Scopus	TITLE-ABS-KEY ("animal-assisted therapy" OR "animal assisted therapy" OR "animal-assisted intervention*" OR "pet therap*" OR "therapy dog*" ) AND TITLE-ABS-KEY ( ICU OR "intensive care" OR "critical care" ) AND TITLE-ABS-KEY ( adult* )

Study of Evidence Selection

**Figure 1:**
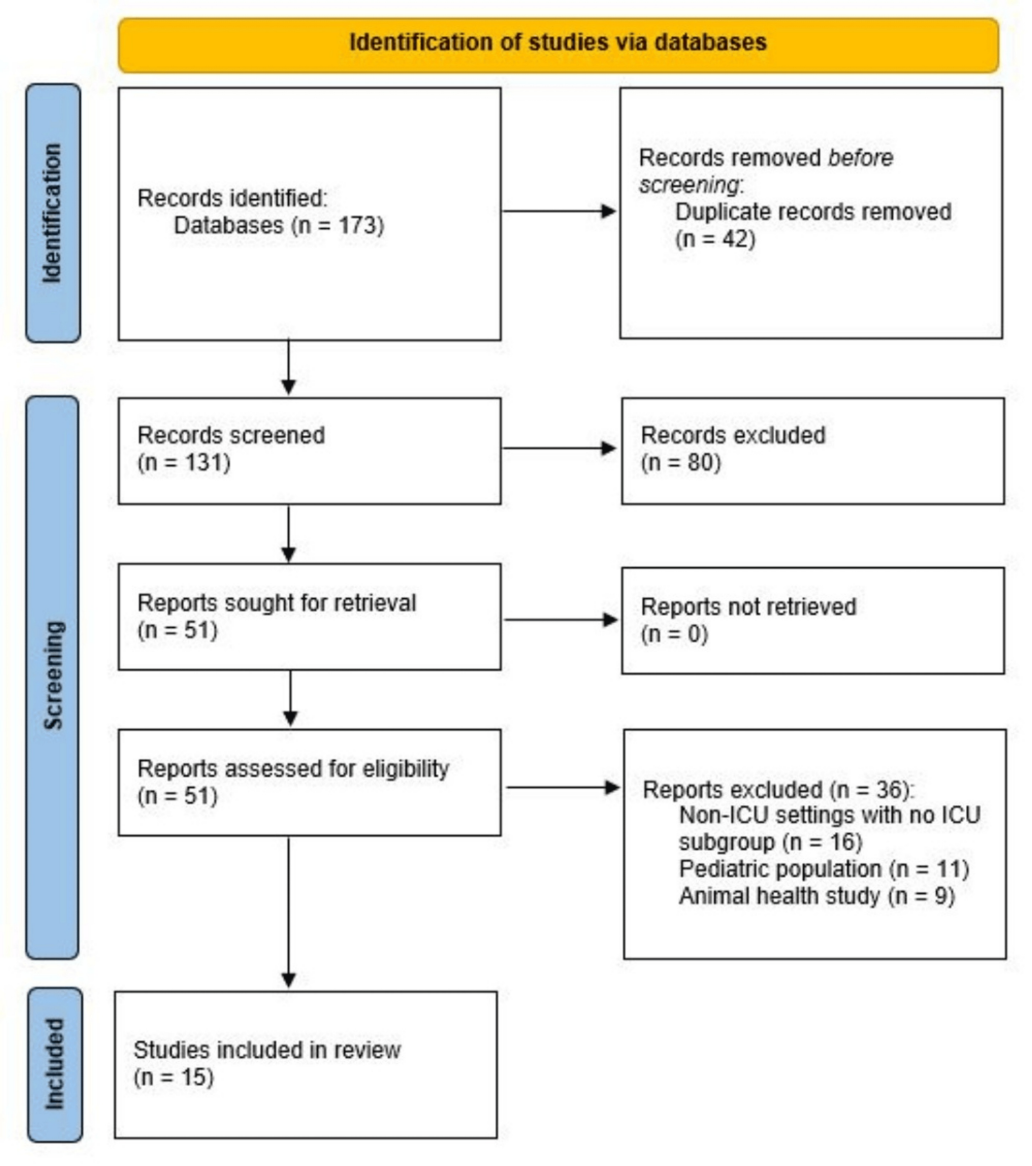
PRISMA flow diagram of the study selection PRISMA: Preferred Reporting Items for Systematic Reviews and Meta-Analyses

All retrieved studies and records were imported into a reference manager, and duplicates were removed. Two reviewers, one who is not a direct author of this scoping review article, independently screened titles and abstracts, and full texts of potentially eligible studies were then assessed. Disagreements were resolved by consensus. The overall search yielded 173 records, and 131 remained after duplicates were removed. On screening by title and abstract, another 80 articles were excluded. This left 51 articles for full-text review. Fifteen studies met the inclusion criteria. The selection process is shown in Figure 1.

Data Extraction

For each included study, a standardized form was used to extract key information and data on the ICU setting and patient population, the animal species and intervention protocol, outcome measures (such as anxiety scales, physiological parameters, and satisfaction surveys), observed results, adverse effects, and noted barriers or facilitators. Because of the heterogeneity of the study design and outcomes, a narrative synthesis was conducted rather than a formal meta-analysis. Two reviewers independently conducted this process to ensure accuracy and consistency, and any discrepancies were resolved through discussion and consensus. Findings were organized into thematic categories, which included anxiety and stress outcomes, pain and physical outcomes, feasibility and safety, and implementation challenges. Insights from review articles and expert commentaries were compared with primary research to identify areas of consensus and discrepancy.

Results

A total of 15 studies were selected for this scoping review (Table 2). The evidence spans from 2015 to 2025 and originates from North America, Europe, and Australia. The study designs were broken down to six empirical studies, five non-empirical studies, and four mixed studies or guideline-based papers. All studies involved certified dogs (or a robotic surrogate) as therapy animals, with no studies using cats or other species. Intervention protocols involved a single session (in research studies) or periodic sessions (weekly or biweekly in service programs) where a handler brought a calm, trained dog to visit a patient at bedside. Session lengths were tailored to patients’ tolerance and ranged from 10 to 30 minutes. Patients were able to interact with the dog by petting, talking, or simply enjoying its presence. Some programs had patients incorporating pets with physical or occupational therapy.

**Table 2:** Selected study articles (n=15) AAI: animal-assisted intervention; PADIS: pain, agitation, delirium, immobility, and sleep disruption; AAA: animal-assisted activities

Article Title	Author(s)	Year	Study Method	Aim	Major Findings
Guidelines for Environmental Infection Control in Health-Care Facilities [6]	Sehulster L, Chinn R	2024	Guideline/reference page	Provide infection-prevention recommendations for animals in healthcare settings	Recommends veterinary oversight, healthy and well-groomed animals, species restrictions, patient screening, and strict hand hygiene to minimize zoonotic risk.
Pawsitive Care: Canine-Assisted Intervention for Anxiety in ICU Patients and Family Members: A Single-Center, Single-Arm Study [7]	Cook KD, Robertson CD, Gudivada KD, et al.	2025	Prospective single-center, single-arm pre–post study	Test whether short therapy-dog visits reduce anxiety in ICU patients and their families	Anxiety decreased immediately after visits for patients and family; no adverse events; possible adjunct effect on pain; calls for longer-term outcomes research.
Risks and Benefits of Animal-Assisted Interventions for Critically Ill Patients Admitted to Intensive Care Units [8]	Fiore M, Cortegiani A, Friolo G, et al.	2023	Systematic review	Summarize the effectiveness and risks of AAI in ICU settings	Six studies were identified, all using dogs; evidence was limited and heterogeneous; no data on survival or infection outcomes; AAI in the ICU was considered experimental pending stronger data.
Robotic Pet Therapy in the Intensive Care Unit [9]	Franck A	2024	Observational program report/quality improvement	Describe the implementation of robotic pet therapy for PADIS-related symptoms in an ICU	Robotic pets were feasible and well-accepted; suggested reductions in agitation and improved comfort; no safety issues were reported.
Animal-Assisted Intervention in the ICU: A Tool for Humanization [1]	Hosey MM, Jaskulski J, Wegener ST, et al.	2018	Editorial/conceptual article	Propose a model for using AAI to reduce suffering and humanize ICU care	Describes psychological burden in ICU and positions AAI as a practical non-pharmacologic strategy; provides principles for program introduction.
Johns Hopkins Brings Therapy Dogs into ICU [3]	Johns Hopkins Medicine	2018	Institutional news release	Announce and contextualize the ICU therapy-dog program	Reports that trained dogs can safely ease physical and emotional suffering and may motivate patients during rehabilitation; outlines the need for clear goals and protocols.
Evaluation of a New Animal Assisted Intervention Service for an Adult Intensive Care Unit [10]	Johnson R	2022	Service evaluation with surveys	Assess the feasibility, safety, and perceived impact of an ICU AAI service	High enjoyment and perceived benefit among patients, visitors, and staff; no adverse incidents; identifies themes such as mood improvement and distraction.
A Multicenter Study of Animal-Assisted Activity and Anxiety Among Older Adults Hospitalized in Acute Care Settings [11]	Kowalski MO, Smith C, Cole DA, et al.	2021	Multicenter interventional pre–post study	Determine whether therapy-dog visits reduce anxiety in hospitalized older adults	Anxiety scores (STAI-6) decreased significantly after visits; this supports AAA as a short-term anxiolytic intervention during hospitalization.
Animal-Assisted Activities in the Intensive Care Unit: A Scoping Review [12]	Lovell T, Ranse K	2022	Scoping review	Map ICU AAA characteristics and risk-mitigation strategies	Six primary ICU studies were identified, mostly with feasibility or observational designs; strong satisfaction was reported; biophysical outcomes were inconclusive due to small samples.
The Impact of an Animal Assisted Activity on Healthcare Worker Well-Being in the Inpatient Hospital Setting [2]	Steinberg B, et al.	2024	Quasi-experimental, waitlist-control staff study	Evaluate effects of therapy-dog sessions on staff stress, burnout, engagement, and mood	No significant changes in stress, burnout, or engagement; immediate improvement in self-reported mood on intervention days; high acceptability.
Animal-Assisted Intervention Services across UK Intensive Care Units: A National Service Evaluation [13]	Wright S, McAree H, Connolly B, et al.	2025	National cross-sectional survey and document review	Determine prevalence, characteristics, and oversight of ICU AAI services in the UK	43% of responding ICUs offered AAI, almost all dog-based; typical frequency weekly - 30–60 minutes; top barriers were infection-control concerns and limited evidence; policies were variable.
Animal-Assisted Interventions for Psychological Distress During Prolonged ICU Stay: A Case Report [14]	Kellogg T, Brockett-Walker C	2025	Case report	Describe the use of weekly animal-assisted interventions to address psychological decline in a lung transplant recipient during a prolonged ICU stay	Patient’s mood and engagement improved markedly, and the intervention was highly acceptable. The case suggests that animal-assisted therapy can effectively enhance psychological well-being and motivation in long-term ICU patients.
Animal-Assisted Interventions in Intensive Care Delirium: A Literature Review [15]	Malik J	2021	Literature review	Ascertain the effect of animal-assisted interventions on critically ill ICU patients, particularly in relation to delirium outcomes	Emerging evidence indicates that animal-assisted interventions can improve ICU care by reducing delirium severity and its related factors, thereby enhancing patient outcomes.
Assisted Animal Interventions in the ICU: We Are Responsible for Ensuring the Well-Being and Ethical Treatment of Animals and Humans [16]	Bocci M, Natalini D, Xhemalaj R, et al.	2024	Correspondence	Highlight the ethical responsibilities and welfare considerations in implementing animal-assisted interventions in ICU settings	Emphasizes that ICU AAI programs must prioritize ethics and safety: involve trained professionals (including veterinarians) to screen for allergies, phobias, or contraindications and follow national guidelines.
Animal-assisted Services in Australia and New Zealand Intensive Care Units: A Cross-sectional Survey [17]	Ranse K, Lovell T, Henderson B, et al.	2025	Cross-sectional survey	Determine the prevalence of animal-assisted services in ICUs across Australia and New Zealand and characterize their practices and barriers	A survey of 63 ICUs found that 44% had offered animal-assisted visits; all programs used dogs with infection-control protocols (e.g., pre-visit bathing, leashing), and no adverse events were reported. Common barriers included a lack of program initiation, staffing constraints, infection control policies, and limited funding.

Patient selection was an important aspect of all pet therapy programs in the studies. Common inclusion criteria were that the patient be awake, alert, and agreeable to an animal visit, with no uncontrolled infection or severe allergies to animals [3]. Many ICUs limited pet therapy to daytime hours when staff could supervise and avoided very critical and unstable patients. The patient populations in these studies ranged from young adults to the elderly with various diagnoses in different ICUs (medical, surgical, cardiac, etc.) [3]. Despite differences in design, the results across these sources showed consistently positive trends in psychological outcomes and high acceptance, with no serious adverse events reported. The sections that follow present findings by outcome domain and summarize reported challenges and implementation experiences. The results measured varied but generally fell into three categories: psychological and emotional outcomes (anxiety, mood, stress), physiological and clinical outcomes (vital signs, pain, some lab stress markers in a few cases), and feasibility/satisfaction, safety, and acceptability outcomes (surveys of patients/families/staff, observation of adverse events).

Psychological and Emotional Outcomes

All studies that evaluated anxiety or stress reported improvements associated with pet therapy. Quantitatively, anxiety scores (measured by visual analog scales or standardized surveys) decreased significantly post-therapy in intervention groups. For example, one study showed patients' median anxiety score fell from 5/10 pre-visit to 0/10 post-visit (p<0.001) [7]. In Kowalski's study, older inpatients had Spielberger State-Trait Anxiety Inventory-6 (STAI-6) anxiety scores drop from a median of 14 to 10 on a 20-point scale (p<0.001) after a dog therapy visit [11]. These studies, along with others, utilized one-group pre/post designs and unanimously found statistically significant reductions in anxiety or distress scales following a therapy dog visit [7,11]. Additionally, family members and caregivers present during ICU pet therapy sessions also experience relief, documenting anxiety decrease from 6 to 3 out of 10, paralleling patients’ improvements [7]. Qualitative comments noted that patients appeared “more relaxed,” “smiled for the first time,” or “seemed less depressed” after interacting with the dog [7,15]. In the 2022 United Kingdom ICU service evaluation, mood elevation was a dominant theme, and patients and visitors described dog visits as uplifting, helping them momentarily forget their illness [13]. Staff echoed that animal-assisted intervention “cheered up the whole unit” and provided positive stimulation for patients who had previously been withdrawn [10,14].

Pain and discomfort levels generally trended downward with pet therapy, although baseline pain was low in some of the ICU cohorts. Cook’s study found a modest but significant reduction in patient-reported pain scores after a therapy dog visit (p<0.001) [7]. In contexts where patients did have notable pain, therapy animals provided distraction and relaxation that improved pain ratings and comfort. For example, John Hopkins' ICU pilot study noted cases where a patient with pain from chest tubes reported feeling better with a dog curled up beside them, attributing the comfort to the animal’s calming presence [3]. None of the ICU studies showed adverse pain effects. At worst, pain remained stable, and at best, it lessened appreciably for some individuals. Beyond pain, sleep and delirium are harder-to-quantify outcomes but were mentioned in some reports. The robotic pet therapy study observed that a few patients (7%) self-reported improved sleep quality with a faux pet in their bed [9]. While data are too limited to draw conclusions on sleep, this hints that the soothing companionship of an animal (even robotic) might ease agitation and promote rest in ICU patients [14,15].

Taken together, psychological outcomes were uniformly positive in the reviewed literature. Pet therapy was associated with less anxiety, more positive mood, high enjoyment, and qualitative reports of reduced stress and loneliness. None of the studies indicated any deterioration in emotional state due to the intervention therapy. Even patients initially skeptical or neutral often warmed up to the therapy animal and expressed gratitude afterward [14]. This strong signal of psychological benefit aligns with decades of research on animal-assisted therapy in other non-ICU healthcare settings. Overall, it suggests that ICU patients, despite their critical illness, are receptive to and can benefit from human-animal interactions.

Physiological and Clinical Outcomes

Results for physiological outcomes (heart rate, blood pressure, respiratory rate, etc.) and concrete clinical endpoints are more mixed and less conclusive with pet therapy intervention. This possibly reflects both the subtlety of these effects and the limited power of existing studies. Several studies measured vital signs before and after dog visits. Most found no significant changes after a pet therapy session. For instance, in one study, heart rate and blood pressure in ICU patients showed no statistically significant difference post-intervention [7]. Coincidentally, this suggests that therapy visits did not cause harmful stress (e.g., no tachycardia or hypertensive spikes), but it also did not dramatically alter or benefit these metrics in a short timeframe. A few individual cases did see mild transient improvements (e.g., a slight drop in blood pressure in a hypertensive patient while petting a dog or a calmer respiratory rate), but these were not consistent enough to rise to significance across groups [7]. Hormonal or biochemical stress markers (like cortisol) were not commonly measured in ICU studies. One earlier non-ICU study found lower cortisol in patients after animal visits, but ICU data on that remain lacking [18]. No ICU-related studies have quantitatively measured whether pet therapy shortens ICU length of stay and days on ventilator or improves mobility scores. These outcomes likely require larger trials. However, qualitative and anecdotal evidence suggest functional gains. As reported by Hosey and others, some ICU patients were motivated to do physiotherapy tasks with a dog present that they might not do otherwise [1,3]. Other examples include patients with prolonged ventilation sitting out of bed and participating in rehab because a small dog was brought in as part of the session, and another bedridden patient standing for several minutes to brush a visiting dog [3]. These reports indicate that animal-assisted intervention may indirectly improve physical outcomes by enhancing patient engagement, even if the immediate physiological effect of a short visit is subtle.

More importantly, there were no negative physiological outcomes attributed to pet therapy. There were no cases of hemodynamic instability, respiratory compromise, falls, or the like reported during supervised animal therapy visits in any study. Patients typically remained medically stable throughout. Some even showed signs of relaxation such as decreased agitation. For example, half the patients in the robotic pet trial had a lower agitation score after spending time with the robotic dog [9]. While not definite, these data suggest that, when proper screening is used to select appropriate patients, pet therapy does not pose a measurable risk to vital sign stability.

Finally, clinical outcomes like infection rates or mortality have not been specifically studied in relation to ICU pet therapy. A systematic review by Fiore noted a complete lack of data on zoonotic infection incidence or survival outcomes in any of the available studies [8]. Additionally, it is not yet known whether animal-assisted intervention might indirectly influence outcomes like delirium incidence, sedation days, or hospital stay length. These topics remain important questions for future research. The absence of infection tracking during animal exposure in studies is notable, given that infection control is a major concern when it comes to animal exposure. As of now, across the available reports, brief animal therapy visits have not been linked to higher infection rates. However, confirming safety will require rigorous, prospective studies with standardized definitions and reporting.

Feasibility, Safety, and Acceptability

Pet therapy was feasible in the ICU settings with appropriate protocols in place based on the reviewed studies and programs. ICUs have successfully integrated therapy dog visits into routine practice on a weekly or case-by-case basis [3,13]. Key findings to determine the feasibility for pet therapy in the ICU included the logistics of bringing in pets (controlling pets with trainers, avoiding interfering with medical procedures, etc.) and whether the ICU environment can accommodate such visits [16]. Johnson’s evaluation explicability concluded that providing animal-assisted intervention in a 20-bed general ICU was feasible and safe, leading to the service’s continuation [10]. The median therapy dog visit in that unit lasted 20 minutes, and staff coordinated to ensure lines and tubes were secure and that the dog had a clear space to interact with the patient. Response rates to post-visit surveys on that project were good, indicating staff were willing to participate in evaluating the service, which is another mark of feasibility [10]. One noted limitation is that not every patient can receive a pet visit on a given day, so scheduling and prioritization are necessary (for example, patients on contact isolation or very unstable were skipped) [16]. In the end, feasible implementation needs to start small and scale up as demand and logistics allow.

No adverse clinical events related to pet therapy were reported across all sources. Specifically, there were no instances of animal bites, scratches, or aggressive behavior in any ICU pet therapy program [7]. Therapy dogs used are carefully temperament-tested, and handlers are trained to read the dog’s stress signals and the patient’s comfort level, intervening early if needed [17]. Likewise, no allergic reactions were documented in the literature, likely because patients with severe pet allergies are excluded by protocol. Infection control monitoring in published pilot studies did not detect any pet-related infections. For instance, Wright noted that infection prevention was a top concern among ICU staff, yet among the ICUs that had pet therapy services, none reported an infection attributed to the therapy animal [13]. Many programs take precautions such as bathing the dog before visits, using clean sheets or barriers on the patient’s bed, and performing hand hygiene for anyone who pets the animal [17]. Measures are taken to mitigate infection risks. The Centers for Disease Control and Prevention's (CDC) guidance by Sehulster on animals in healthcare indicates that dogs and cats can carry zoonotic pathogens, but with proper precautions, the risk of transmission is low [6]. The current evidence on safety is largely reassuring, though admittedly based on relatively few cases. As Fiore concluded, the absence of observed harms is encouraging, but formal data on safety endpoints are “scarce” and pet therapy in the ICU should still be considered somewhat experimental until further data are available [8]. Nonetheless, the consistent reporting of “no adverse events” in existing studies is a strong positive signal that pet therapy is safe in the ICU setting.

Finally, there are high acceptability and satisfaction rates when it comes to pet therapy. Patients overwhelmingly responded positively to any pet therapy opportunities. In a United Kingdom survey evaluation, 83% of patients and visitors responded with a 10/10 in enjoyment rating when it came to dog visits [13]. In Wright’s survey, ICUs with animal-assisted therapy programs reported strong support for the service by patients and families [13]. Among available studies, patients, family members, and clinicians consistently report strong satisfaction and support for animal-assisted activities [12,14]. Staff attitudes tend to become favorable after seeing the benefits and lack of disruptions during pet therapy sessions. The qualitative study by Hosey described that, anecdotally, a dog sitting on a patient’s lap eases suffering and helps in ways medical intervention may not, which further can motivate a clinician’s view on pet therapy as an adjuvant treatment [1]. Even at a systems level, there is growing endorsement of animal-assisted intervention. For example, the Intensive Care Society in the United Kingdom has published guidance encouraging ICUs to consider animal-assisted intervention as part of holistic care [13]. Taken together, these findings indicate that animal-assisted intervention is well-accepted by patients, families, and staff and, with appropriate infection control and governance, merits structured integration as an additional modality to ICU care.

In summary, the results from the reviews indicate that pet therapy in adult ICUs is generally feasible to implement, safe in practice, and highly acceptable by patients, families, and staff. The interventions lead to consistent improvements in psychological well-being (anxiety, mood, etc.) and are met with enthusiasm. Hard physiological or clinical outcome benefits are less certain with current data, but there are promising signs (like increased engagement and motivation in patients with no adverse effects) that justify continued use and further study.

Discussion

The synthesis of current literature reveals that pet therapy is a promising adjunct for improving the ICU patient experience, though it remains an evolving practice supported by modest evidence. This section will continue to review the emerging evidence on animal-assisted intervention, evaluating the psychological, physical, and rehabilitation outcomes, while incorporating the perspectives of patients, families, and staff, and appraising the current program models of pet therapy. It will also analyze the reported results and outline some of the practical and ethical challenges of pet therapy in the adult ICU.

It is important to recognize that pet therapy is not a stand-alone treatment for medical issues, but rather a complementary intervention. Its strength lies in addressing the psychological and emotional dimensions of critical illness, which are often underscored by pharmacologic treatments. In the holistic ICU care model, interventions like animal-assisted therapy, music therapy, relaxation techniques, etc., form a toolkit to reduce suffering and improve patient participation [2,11]. Pet therapy is one of the more engaging tools because it provides interactive and personalized stimulation, which is hard to achieve otherwise in an ICU room [15]. Because of the minimal risks, pet therapy is just another option to approach caring for critically ill patients as a whole.

The principal takeaway is that animal-assisted therapy can humanize the ICU and provide tangible psychological benefits for patients. ICU survivors often suffer from anxiety, depression, and post-intensive care syndrome symptoms; reviewed studies suggest therapy animals can help alleviate and mitigate symptoms during the ICU stay [11,15]. Even a short interaction with a therapy dog offered comfort, reduced feelings of loneliness, and made the ICU environment feel less frightening. This aligns with theories in human-animal interaction research, which propose that positive contact with animals can trigger neurophysiological responses (such as oxytocin release) and reduce cortisol, which can lead to decreased stress and improved mood [2]. While ICU-focused biochemical studies are lacking, it can be reasonable to extrapolate some of these mechanisms to critically ill patients. Another valuable application in pet therapy is in motivation and rehabilitation. Therapy dogs serve as a form of psychological support that can motivate patients to participate in their care [15]. The concept of “motivator dog” in ICU rehabilitation emerged in the commentary by Hosey [1]. This concept is also echoed by clinicians who observed patients achieving physical therapy goals with the dog’s encouragement [3]. This suggests that pet therapy might be strategically used alongside early mobility programs to enhance patient engagement. Additionally, some ICUs have utilized therapy dogs to assist in breaking severe ICU delirium or dissociation, for example, by giving a delirious patient a simple, calming focus (the presence of a dog), which may help reorient them. Data on this is something that needs to continue to be explored.

Given the short duration of most animal-assisted intervention sessions, large impacts on physiological or clinical outcomes are less evident, yet some encouraging observations have been made. Though vital signs do not drastically change during a pet therapy visit, it also shows there are no signs of destabilization leading to any harm for patients [7,14]. There have been no multi-center studies to date demonstrating improvements in ICU mortality, length of stay, or other negative outcomes attributable to pet therapy [8]. Though objective physical metrics (like ventilator time or muscle mass) have not been rigorously studied with pet therapy, functional benefits are plausible and can be a point for further research. It has been anecdotally seen that pet therapy leads to improved engagement and activity, which can translate to maintenance of muscle strength and orientation, which are critical for ICU rehabilitation. Animal-assisted intervention has been seen to “promote recovery behavior” in the ICU and even help ICU patients mobilize better with a dog present [1,3]. Though there have been no studies done for survival rates and outcomes, there have been none conducted for infection rates as well [8,12]. Overall evidence of effectiveness remains limited and inconclusive, but standardized screening, infection prevention practices, trained handlers, and protocolized ICU workflows for animal-assisted intervention appear to minimize any risks.

On the implementation side, sharing best practices and developing standardized guidelines for pet therapy will be important. Efforts like the Royal College of Nursing’s protocol and the Intensive Care Society guidance serve as blueprints for hospitals to adopt pet therapy safely [13]. Further national guidelines and systemized protocols for how to handle pet therapy in hospitals can mitigate concerns and risks for things such as infection [6]. Despite the implementation of protocols and guidelines and the growing evidence of benefits, pet therapy is not yet standard practice in most adult ICUs. A national survey in ICUs in the United Kingdom found that 43% of responding ICUs offered some form of animal-assisted intervention [13]. The majority of those had informal animal-assisted intervention programs (friendly pet visits for comfort), and only a couple had structured animal-assisted therapy programs with specific clinical goals [13]. Importantly, the survey did highlight that infection concerns and lack of evidence were the most frequently cited barriers in ICUs that did not have animal-assisted intervention services [13]. Though many critical care units remain cautious, pilot programs have shown that implementation is feasible. Johnson’s study evaluated a new ICU therapy dog service in an English hospital and reported no adverse incidents over its trial period [10]. Key elements of successful programs include close collaboration with volunteer handler organizations, careful patient selection, and strict protocols for animal health and hygiene [3,10]. Standardized guidelines from protocols mentioned above, such as requiring therapy animals to be well-groomed, vaccinated, and certified, also minimize infection risks under supervised conditions [6,13]. With safeguards in place, studies have shown that there is no reported physical harm, such as dog bites, scratches, or infections [7,17]. With this in mind, pet therapy can be implemented and utilized to become a goal-directed service as long as standardization and guidance is followed.

Despite its promises, implementing pet therapy in an ICU comes with notable challenges that must be managed. Infection control is a dominant concern because ICU patients can be immunocompromised or have invasive lines and wounds. The CDC guidance recognizes animals as potential vectors, carrying organisms such as Salmonella, in healthcare settings, so successful programs use strict protocols, such as verified vaccinations and regular veterinary screenings, pre-visit bathing and grooming, and rigorous hand hygiene [6]. Allergies and phobias to animals require opt-in consent, screening, staff notification, and, when appropriate, use of hypoallergenic breeds with limited contact. Fortunately, there have been no studies reporting any allergic reactions due to animal-assisted intervention. Concerns for animal behavior and welfare call for certified dogs, trained handlers, and capped visit frequency. Because of this, there have been no reported ICU episodes described as aggressive incidents [7]. Operational hurdles include obtaining institutional approval, drafting policies, allocating staff time, and coordinating around procedures, yet pilot programs with a designated pet therapy unit have shown that visits can fit safely into workflow [17]. Finally, because critical care medicine is strongly evidence-based, the adoption of animal-assisted intervention may be limited until further studies emerge. Until stronger evidence develops, cautious, protocol-driven implementation that prioritizes safety and patient preference is reasonable. While there are some challenges to pet therapy, a well-designed and protocol-driven program remains a safe, patient-centered adjunct that can support engagement in ICU care.

In the past 10 years, investigators have gradually started evaluating animal-assisted interventions in adult ICUs. Early evidence was limited to case reports and anecdotal descriptions, but more formal studies have been conducted in recent years, including observational trials, program evaluations, and a few controlled studies. A 2022 review by Lovell and Ranse identified only six primary research studies on ICU animal-assisted activities worldwide [12]. Notably, there are no studies done on animal-assisted intervention from other species (such as cats or rabbits), with most published data focusing on therapy dogs, likely due to the dogs’ trainability and patient acceptance [8]. As holistic care becomes more utilized in the ICUs to assist with patients’ treatment, there will be a continued push for studies on pet therapy. Future research will consist of larger controlled trials that study the physiological and clinical benefits while also tackling safety more in-depth from pet therapy. Studies on other animals or different formats (for example, virtual pet therapy) can also be considered. Finally, a comprehensive way to assess the impact on the patient, family, and staff is needed. Though early evidence shows family and staff do benefit from pet therapy, there needs to be a more systematic way to analyze its effects through standardized outcome measures, consistent safety reporting, and adequately powered controlled studies [7,9]. There are a lot of other additional future directions research can dive into when it comes to pet therapy. Though studies have been conducted to see if any animal-assisted intervention leads to benefits, there are still various results and long-term outcomes that still need to be answered. Measurable outcomes, such as ventilator days, ICU course, and physical improvement, can be limited due to the nature of the therapy, but may be possible one day. Other avenues for future studies could assess if daily therapy dog visits over a week lead to a sustained reduction in anxiety or improved psychological states at discharge. Additionally, long-term outcomes (for example, does ICU pet therapy reduce post-traumatic stress disorder or depression after ICU discharge) is another interesting area for exploration. Currently, no studies have that follow up, which suggests researching the influence of long-term psychological outcomes from pet therapy is important [7]. Overall, as data continues to grow, pet therapy will be increasingly integrated into ICU practice as an evidence-based, patient-centered adjunct.

Finally, the move toward a more holistic, humane care in intensive care medicine is at the forefront. Patients are not simply problems to be medically managed. They are humans undergoing frightening, painful experiences. Interventions like pet therapy acknowledge the personhood of the patient, offering empathy, comfort, and a form of emotional therapy [16]. In an ICU, a patient’s psychological needs, along with physiological needs, should be met. Pet therapy, when done safely, can be seen as an ethical practice to reduce suffering. Of course, the ethical use of animals should also be considered. Therapy animals and their handlers are typically volunteers “working” out of goodwill. They should be treated well and not put in harmful situations. Given that therapy dogs generally enjoy social interaction, the arrangement can be mutually beneficial [11]. Ensuring the animal’s welfare is part of the ethical implementation as well [16]. When programs protect both patients and animals, pet therapy becomes a humane, patient-centered adjunct to ICU care that can reduce suffering and uphold the dignity of the patient.

In conclusion, the discussion highlights that pet therapy holds real value in the adult ICU as a means of humanizing care and supporting patients’ mental health. Still, it must be implemented thoughtfully. Challenges like infection control and limited evidence can be overcome with rigorous protocols and ongoing research. As more data emerges, pet therapy could transition to a standard component of comprehensive ICU care. With the positive experiences thus far, along with the paradigm shift in ICU patient care, something as simple as a dog’s companionship might have a profound impact on the fast-paced world of critical care, providing a dose of humanity in even the most dire moments.

## Conclusions

This scoping review shows the feasibility and benefits of pet therapy as a complementary intervention in adult ICU settings. It has moved from a novel idea to a practical reality in many hospitals over the last decade, bringing the healing presence of animals into the realm of critical care. This review has outlined how, with proper safeguards, animal-assisted intervention can be applied in adult ICUs to improve patient outcomes in terms of anxiety, mood, and overall experience. Despite challenges, the emerging findings portray pet therapy as a low-risk, high-reward intervention to help address the psychological challenges of critical illness. There are significant opportunities for future research, including examining the impact of pet therapy on delirium incidence, duration of sedation, and hospital length of stay. Further studies in these areas would help clarify the broader clinical effects of pet therapy and strengthen the evidence base for their integration into ICU care. In conclusion, pet therapy offers a powerful means to humanize intensive care. ICU pet therapy is not just about making patients happy in the moment. It is about addressing the fundamental human need for comfort and connection amid intensive medical treatment. With careful implementation, pet therapy can be a safe, low-cost, and effective tool to improve the holistic well-being of ICU patients and their families.
